# LEDPatNet19: Automated Emotion Recognition Model based on Nonlinear LED Pattern Feature Extraction Function using EEG Signals

**DOI:** 10.1007/s11571-021-09748-0

**Published:** 2021-11-25

**Authors:** Turker Tuncer, Sengul Dogan, Abdulhamit Subasi

**Affiliations:** 1 Department of Digital Forensics Engineering, Technology Faculty, Firat University, Elazig, Turkey; 2 Institute of Biomedicine, Faculty of Medicine, University of Turku, 20520 Turku, Finland; 3 Department of Computer Science, College of Engineering, Effat University, Jeddah, 21478 Saudi Arabia

**Keywords:** Led-pattern, TQWT, S-Box based feature generation, RFIChi2, Emotion recognition, Machine learning, Artificial intelligence

## Abstract

Electroencephalography (EEG) signals collected from human brains have generally been used to diagnose diseases. Moreover, EEG signals can be used in several areas such as emotion recognition, driving fatigue detection. This work presents a new emotion recognition model by using EEG signals. The primary aim of this model is to present a highly accurate emotion recognition framework by using both a hand-crafted feature generation and a deep classifier. The presented framework uses a multilevel fused feature generation network. This network has three primary phases, which are tunable Q-factor wavelet transform (TQWT), statistical feature generation, and nonlinear textural feature generation phases. TQWT is applied to the EEG data for decomposing signals into different sub-bands and create a multilevel feature generation network. In the nonlinear feature generation, an S-box of the LED block cipher is utilized to create a pattern, which is named as Led-Pattern. Moreover, statistical feature extraction is processed using the widely used statistical moments. The proposed LED pattern and statistical feature extraction functions are applied to 18 TQWT sub-bands and an original EEG signal. Therefore, the proposed hand-crafted learning model is named LEDPatNet19. To select the most informative features, ReliefF and iterative Chi2 (RFIChi2) feature selector is deployed. The proposed model has been developed on the two EEG emotion datasets, which are GAMEEMO and DREAMER datasets. Our proposed hand-crafted learning network achieved 94.58%, 92.86%, and 94.44% classification accuracies for arousal, dominance, and valance cases of the DREAMER dataset. Furthermore, the best classification accuracy of the proposed model for the GAMEEMO dataset is equal to 99.29%. These results clearly illustrate the success of the proposed LEDPatNet19.

## Introduction

### Background

The development in computer technology has led to growth in the global gaming market. The gaming industry is widely serving people with its developing graphic and sound infrastructure (Chanel et al. 2011; Vasiljevic and de Miranda 2020). People play computer games for different purposes such as entertainment and learning. At the same time, computer games are also used in researches for determining the emotional states of people to understand enjoyable level of the game (Dasdemir et al. 2017). Computer games have different effects on the participants such as funny, boring, horror, calm. Researchers use Brain-Computer Interfaces (BCI) to monitor these effects and they especially have used EEG signal to understand these effects (Bigirimana et al. 2020; Djamal et al. 2020; Vasiljevic and de Miranda 2020). BCI is a tool that enables interaction between people and computer systems (Pan et al. 2013). Especially, wearable devices help to develop neuroscience and neurogaming platforms. Moreover, BCIs have been used to understand the collected signals (Parsons et al. 2020; Reuderink et al. 2009). Conventional methods such as surveys and interviews are also used to determine the impact of a game on users. However, this method does not always reach the correct result. Modern systems need internal parameters, and lower margin of error. EEG signals are used in the systems with BCI to monitor the effects of a game in the human mind. Moreover, EEG signals reveal the electrical activity of neurons in the brain. Thus, measuring this electrical activity provides experts with an opinion on assessing brain activity (Rahman et al. 2020; Ullal and Pachori 2020). The effects of computer games on different people can be evaluated with EEG signal analysis. Moreover, the development of games can be provided by following the effects of a game on people such as boring, calm, and horror. Thus, computer games released in the game market can be improved. People’s reactions to different situations can be measured by using game applications, and more realistic systems can be created (Bharti and Patel 2020; Gaume et al. 2019; Manshouri et al. 2020; Miah et al. 2020).

### Motivation

Computer games have a huge market, and many people play several games. Computer game producers/developers want to know feedbacks about their games. Therefore, many surveys have been applied to players. However, true feedbacks cannot be achieved by using these surveys. Thus, feedbacks must be collected during the game. One of the feedback collection models is EEG based emotion recognition.

Our main motivation is to identify emotions using EEG signals but emotion recognition using EEG is one of the complex issues of machine learning. Many models have been presented in the literature to recognize/classify emotions with high classification accuracy. The primary objective of the presented model is to propose a highly accurate emotion recognition model using EEG signals. Therefore, a novel hand-crafted feature extraction network is presented. This network aims to extract low-level, high-level, and medium-level features. The other aim of this method is to demonstrate the success of cryptologic structures in feature generation. Therefore, an S-box based textural feature generation function is presented and named as Led-Pattern. By using the proposed LED pattern, hidden features/patterns in the EEG signals can be detected easily and a high accurate EEG signal recognition model can be proposed by using the hidden nonlinear patterns. By using the presented model, emotions can be classified with high accuracy. New generation intelligent emotion recognizers can be developed by implementing the presented Led-Pattern and RFIChi2 (Tuncer et al. 2020). Moreover, two EEG datasets have been used to denote the general success of the proposed LEDPatNet19.

### Related works

In this section, we presented some of the EEG based emotion recognition studies. Alakus et al. (Alakus et al. 2020) created an emotion database using EEG signals. In the study, emotional states were recorded with 4 computer games. EEG signals were collected by allowing users a total of 5 min for each game in the study. Rejer et al. (Rejer and Twardochleb 2018) suggested a model to monitor the effect of games on the brain. Different games were selected for the participants for this purpose. The proposed method is based on genetic algorithm. With the proposed method, it is determined which part of the brain is effective in the game. Hazarika et al. (Hazarika et al. 2018) proposed a method for the inhibitory control function. Action video game is used for this purpose. EEG data of 35 players were used in the study. Alpha, beta, and gamma frequencies of EEG signals are taken into account. This study was based on Discrete Wavelet Transform, and SVM was chosen as a classifier. 98.47% accuracy rate was obtained in the study. Shih-Ching et al. (Yeh et al. 2018) presented a gaming platform using a brain-computer interface and virtual reality. EEG and EMG signals are used together to create this gaming platform. Manshouri et al. (Manshouri et al. 2020) suggested a method to test the EEG signals of users watching 2D and 3D movies. Also, EEG signals were measured and recorded before watching movies. The same operations were carried out after watching the movie. Thus, the effects of these films on brain activity and power spectrum density were observed. This study was based on short-time Fourier transform and achieved 91.83% accuracy. Parsons et al. (Parsons et al. 2020) proposed a classification method using the EEG signals. This study used video game player experiences. EEG signals were collected from 30 participants for this purpose. Support Vector Machine, Naïve Bayes, k-Nearest Neighbors were used as classifiers. Naive Bayes was identified as the best classifier for the negative game platform. K-Nearest Neighbors has provided more successful classification results on general gaming platforms. Alchalabi et al. (Alchalabi et al. 2018) introduced a method to detect patients with attention deficit hyperactivity disorder. For this purpose, the distinctive features of EEG signals are used. Besides, healthy and attention deficit hyperactivity disorder patients were evaluated together. The condition classification accuracy rate was calculated as 96.0% in healthy persons. In attention deficit hyperactivity disorder patients, accuracy was 98.0%. Scherer et al. (Scherer et al. 2013) proposed a model for functional brain mapping. The proposed method is based on Kinect-based games. Thus, the reactions of people to these games were evaluated with EEG signals. Chanel et al. (Chanel et al. 2011) presented an approach aimed at gathering information about the game using surveys, EEG signals, and environmental factors. In this study, the Tetris game was used for three difficulty levels. Furthermore, it was determined that different levels of difficulty could be distinguished using these parameters. The accuracy rate was calculated as 63.0%.

### Proposed approach

The proposed EEG based emotion recognition model has preprocessing, feature generation, feature selection, and classification phases. In the preprocessing phase, the loaded EEG signals are divided into frames for the GAMEEMO dataset since GAMEEMO dataset lengthy EEG signals. Then, the preprocessed EEG signals are fed to TQWT (Hussain 2018; Wang et al. 2014) for sub-bands generation. For this work, 18 sub-bands have been generated to generate features at high level since TQWT is an effective one-dimensional signal transformation model. The presented Led-Pattern and statistical feature generation function extracts 540 features (Led-Pattern extracts 512 features, and 14 statistical features are generated from the raw EEG signal and decomposed EEG signal sub-bands). The used feature generators extract 540 features from 19 signals (18 sub-bands and a raw EEG signal). Thus, the presented learning model is named LEDPatNet19. These extracted features are concatenated, and RFIChi2 is applied to the concatenated feature vector to select the most informative ones. In the classification phase, support vector machine (SVM) classifier has been utilized (Hassoun 1995; Park et al. 1991) (Glorot and Bengio 2010; Yosinski et al. 2014).

### Novelties and contributions

Novelties of the proposed Led-Pattern based model:

- A nonlinear one-dimensional textural feature generation model is presented by using S-box of the Led cipher algorithm (Led-Pattern) (Kushwaha et al. 2014; Mendel et al. 2012).
- By using TQWT (Hussain 2018; Wang et al. 2014), Led-Pattern, and statistical moments, a new generation, lightweight and multilevel feature generation model is presented. Herein, the effective models are used together. TQWT is an effective transformation model to obtain wavelet frequencies of the used signal. By using these frequency sub-bands, features at high level are extracted using Led-Pattern (textural features) and statistical moments (statistical features). RFIChi2 is a hybrid selector and selects the most informative features. By using SVM (it is an effective shallow classifier), classification results are obtained.
- Contributions of the proposed Led-Pattern based model:
- S-boxes have generally been utilized for nonlinear transformations. Therefore, many block cipher algorithms have been used S-boxes. This work aims to discover the effect on the S-boxes for feature generation. Two of the widely preferred hand-crafted methods are textural and statistical feature generators. In the statistical feature generation models, statistical moments have been used. While textural feature generators use variable linear patterns for local feature generation to extract global optimum features like binary pattern. The proposed Led-Pattern uses a nonlinear pattern (S-box of the Led cipher). The feature generation capability of a nonlinear textural feature generation model has been demonstrated, and a new feature of S-Boxes has been shown.
- In order to achieve high classification performance for EEG based emotion recognition during game playing, a cognitive problem–solution methodology is used in this research. TQWT, statistical features, and Led-Pattern are used together to generate effective features. RFIChi2 selects optimal features. The proposed LEDPatNet19 achieved high classification performances on the used two EEG datasets. In this respect, the LEDPatNet19 is a high accurate model.

## Material

We have used two datasets to denote success of the developed LEDPatNet19 and these datasets are GAMEEMO and DREAMER. These two datasets were collected from EMOTIV EEG brain cap and EMOTIV software were applied these signals for preprocessing. More details about these datasets are given below.

### GAMEEMO

In this study, a database called GAMEMO created by Alakus et al. (Alakus et al. 2020) was used. This is a publicly available database. This database was created by collecting EEG signals via the 14 channel EMOTIV EPOC+ (wearable and portable) device. The sampling rate of these EEG signals is 128 Hz (2048 Hz internal) and a has a denoising filter. By using this filter, the bandwidths of the used EEG signals are calculated as 0.16 Hz and 43 Hz. 28 different subjects were used in the created database. These subjects played four different games. These games were calm, boring, funny, and horror. Each individual was allowed to play each game for a total of 5 min. Thus, 20 min EEG data of each individual were collected. These data were in csv and mat formats. The reactions of the subjects to the games were monitored and recorded using these games. In addition, Self-Assessment Manikin (SAM) form and rating scores were obtained for each subject. The purpose of creating a SAM form in this study is to rank each game according to the scale of arousal and valence. In this dataset, the length of each EEG signal is equal to 38,252. Therefore, we divide these signals into fixed-size non-overlapping segment with a length of 7650.

### DREAMER

Dreamer dataset is a commonly used EEG emotion datasets in the literature and has three cases. These cases are named arousal, dominance and valance. The used each case contains two classes and they are low and high. The DREAMER dataset was collected from 23 participants using an EMOTIV EPOC wearable EEG device. By using this device, EEG signals have been collected with 14 channels like GAMEEMO dataset. To collect the emotional EEG signals from these 23 participants, 18 film clips are selected with a length range of from 65 to 393 s. 128 Hz sampling rate was set to collect EEGs. Moreover, band-pass frequency filter [4–30 Hz] was used to denoise artefacts.

## The proposed Led-Pattern algorithm

**Fig. 1:**
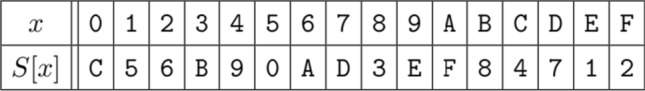
S-box of the Led cipher

This research suggests a new generation binary pattern (BP) like nonlinear textural feature generation function. As stated in BP, it considers a linear pattern to generate features. The presented Led-Pattern uses an S-box of a lightweight block cipher, which is Led cipher. Led cipher has a 4-bit S-box, and is shown in Fig. 1 (Kushwaha et al. 2014; Mendel et al. 2012).

**Fig. 2:**
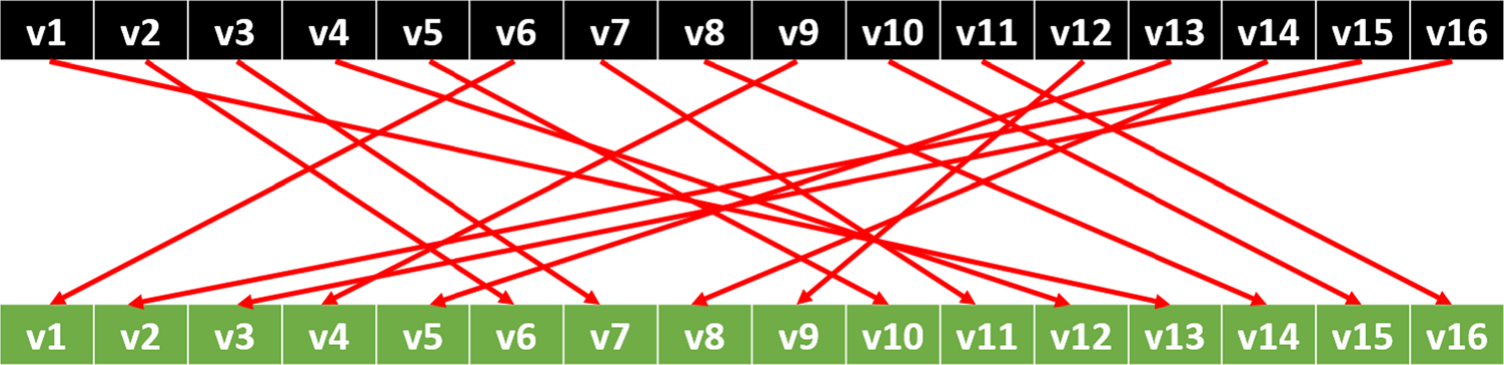
The pattern of the Led-Pattern. Herein, v1, v2, …, v16 define values of the used overlapping block with a length of 16

Figure 1 shows the values of this S-box. By using this nonlinear structure, a nonlinear pattern is created. As seen from Fig. 1, the length of this S-box is 16. Therefore, feature generation is processed on the 16 sized overlapping blocks. xth values and S[x]th values are compared to generate bits. The graphical expression of the used pattern is also shown in Fig. 2.

The steps are given below for the better understanding of the proposed Led-Pattern.

- *Step 1*: Apply overlapping block division to a one-dimensional signal. Here, the length of the block is selected as 16.
  1 $$blc=signal\left(i:i+15\right), i=\left\{\mathrm{1,2},\dots ,L-15\right\}$$
  where $blc$ and $L$ define 16 sized blocks and length of the signal.
- *Step 2*: Generate bits by using the presented nonlinear pattern, which is shown in Fig. 1.
  2 $$bit\left(k\right)=sgnm\left(blc\left(x\left(k\right)\right),blc\left(s\left[x\right]\left(k\right)\right)\right), k=\{\mathrm{1,2},\dots ,16\}$$
  3 $$sgnm\left(blc\left(x\left(k\right)\right),blc\left(s\left[x\right]\left(k\right)\right)\right)=\left\{\begin{array}{c}0, blc\left(x\left(k\right)\right)-blc\left(s\left[x\right]\left(k\right)\right)<0\\ 1, blc\left(x\left(k\right)\right)-blc\left(s\left[x\right]\left(k\right)\right)\ge 0\end{array}\right.$$
- $sgnm(.,.)$ is signum function, and it is utilized for primary bit generation function.
- *Step 3*: Construct left and right bit groups using the extracted 16 bits.
  4 $$bl\left(j\right)=bit\left(j\right), j=\{\mathrm{1,2},\dots ,8\}$$
  5 $$bt(j)=bit(j+8)$$
  where $bl$ and $br$ are left and right bits groups, respectively.
- *Step 4*: Generate left and right signals.
  6 $$left(i)=\sum_{j=1}^{8}bl\left(j\right)*{2}^{j-1}$$
  7 $$right(i)=\sum_{j=1}^{8}br\left(j\right)*{2}^{j-1}$$
- Both signals which are right and left are coded in 8-bits. Hence, the length of histograms of each signal is calculated as ${2}^{8}=256$.
- Step 5: Extract histogram of the left (${hist}^{left}$) and right (${hist}^{right}$) signals.
  8 $${hist}^{left}\left(j\right)=0, j=\{\mathrm{1,2},\dots ,256\}$$
  9 $${hist}^{left}\left(j\right)=0$$
  10 $${hist}^{left}\left(left\left(i\right)\right)={hist}^{left}\left(left\left(i\right)\right)+1, i=\{\mathrm{1,2},\dots ,L-15\}$$
  11 $${hist}^{right}\left(right\left(i\right)\right)={hist}^{right}\left(right\left(i\right)\right)+1$$
  Step 6: Concatenate histogram extracted to obtain features of Led-Pattern (${f}^{Led-Pat}$).
  12 $${f}^{Led-Pat}\left(i\right)={hist}^{left}\left(i\right), i=\{\mathrm{1,2},\dots ,256\}$$
  13 $${f}^{Led-Pat}\left(i+256\right)={hist}^{right}\left(i\right)$$

As stated Eqs. 12, 13, the proposed Led-Pattern generates 512 features from a signal.

Steps 1–6 consist of the presented Led-Pattern feature generation function, and it is called as $led-pat(.)$ in the proposed classification model to better expression.

## The proposed led-pattern based emotion recognition model

**Fig. 3:**
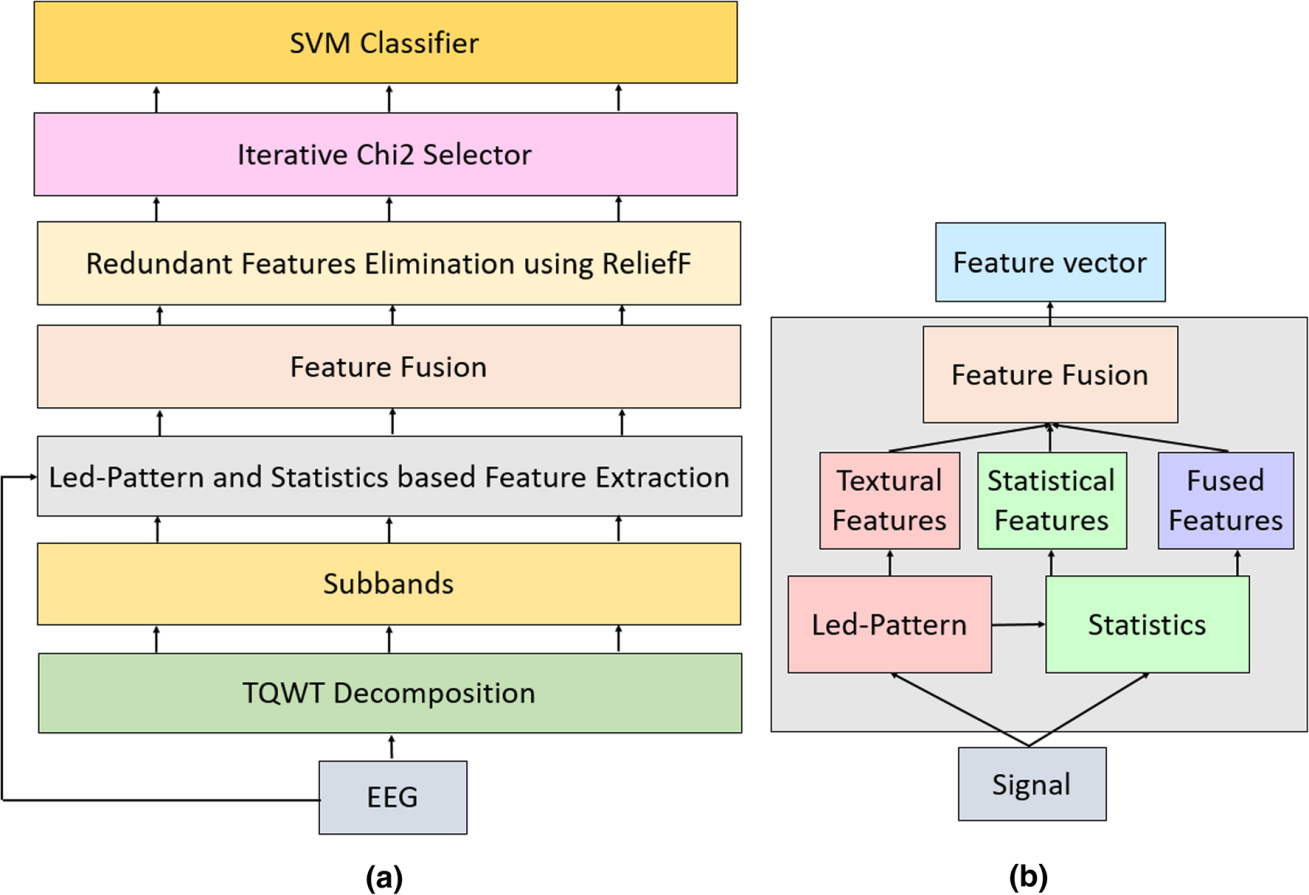
Schematic explanation of the proposed LEDPatNet19 **a** graphical overview of the proposed model, **b** the proposed fused feature extractor

This work presents a new generation EEG signal classification model to recognize emotion. The presented model aims to achieve high-performance for emotion recognition using EEG signals. A new cognitive model is presented using the effectiveness model, which is handled in four phases. These phases are preprocessing (framing), feature generation using TQWT and fused feature extraction function (Led-Pattern and statistical feature extraction), selection of the optimal features with RFIChi2 feature selector, and classification using SVM phases. The most important phase of the proposed model is feature extraction. In the feature extraction phase, TQWT creates 18 sub-bands by using 2,3,17 Q-factor, redundancy and number of levels parameters. The used hybrid generator (Led-Pattern and statistical extractor) extracts 540 features from raw EEG signal and 18 sub-bands. Thus, this model is called LEDPatNet19. A schematic overview of this model is shown in Fig. 3 for better understanding.

Figure 3 summarizes our proposed LEDPatNet19. In the first phase, TQWT is deployed to the EEG signal. However, we used fixed-size non-overlapping blocks to increase the number of observations. In this work, Q-factor (Q), redundancy (r), and the number of levels (J) parameters are chosen as 2,3, and 17 respectively. Thus, 18 wavelet sub-bands are created. The presented Led-pattern based fused feature extractor (see Fig. 3b) extracts 540 features from each signal. Herein, 19 signals are utilized as input of the proposed fused feature extractor. In the feature fusion/merging phase, the extracted 19 feature vectors with a length of 540 are combined and a final feature vector with a length of 540 × 19 = 10,260 is created. RFIChi2 feature selector has been used for this work. The main purpose of this selector is to use the effectiveness of both ReliefF and Chi2 selectors. ReliefF selector can generate both negative and positive weights for each feature. The negative weighted features can assign redundant features. Chi2 is one of the fastest selectors but it cannot select the best feature vector automatically. Therefore, iterative Chi2 has been used in the second layer of RFIChi2. The chosen features are classified using an SVM classifier with tenfold cross-validation. The general steps of the proposed LEDPatNet19 are:

- *Step 1:* Apply TQWT to each frame/EEG signal.
- *Step 2:* Use the fused feature generation model and extract features from the original EEG frame and decomposed signals.
- *Step 3:* Select the most informative features from the feature vector extracted by using RFIChi2 feature selector.
- *Step 4:* Classify these features using SVM.

More details about the presented LEDPatNet19 are given below.

### Fused feature generation model

The first phase is feature generation. To generate effective features, both textural and statistical features are used together. Both statistical and textural feature generation functions have been widely preferred in hand-crafted feature generation. Therefore, we used both of them. The used textural feature generator is Led-Pattern, and it was defined in Sect. 3. It is a histogram-based feature generator, and it generates 512 features from an EEG signal. 14 statistical moments have been chosen to generate statistical features. Also, a decomposition method is used for multilevel feature generation. This decomposition method is TQWT, which is one of the new generation decomposition techniques. It is an improved version of the single-level Q-factor wavelet transform and takes three parameters. These parameters are Q-factor, r (redundancy level), and the number of levels (J). Eighteen levels wavelet transformation is used by deploying 2,3 and 17 Q, r, and J parameters, respectively.

The used statistical feature generation function was demonstrated by using $ist(.)$. The statistical moments which are consisted of $ist(.)$ feature generation function are shown as below.

14 $$fst\left(1\right)=\frac{1}{M}\sum_{i=1}^{M}sgnl(i)$$

15 $$fst\left(2\right)=\sqrt{\frac{1}{M-1}\sum_{i=1}^{M}{\left(sgnl\left(i\right)-fst\left(1\right)\right)}^{2}}$$

16 $$fst\left(3\right)=\sum_{i=1}^{M}sgnl(i)$$

17 $$fst\left(4\right)=-\sum_{i=1}^{M}\frac{sgnl\left(i\right)}{\sqrt{\frac{\sum_{i=1}^{N}sgnl{\left(i\right)}^{2}}{M}}}log\frac{sgnl\left(i\right)}{\sqrt{\frac{\sum_{i=1}^{N}sgnl{\left(i\right)}^{2}}{M}}}$$

18 $$fst\left(5\right)=\frac{1}{M}\sum_{i=1}^{M-1}|sgnl\left(i+1\right)-sgnl\left(i\right)|$$

19 $$fst(6)=\frac{\sqrt{M(M-1)}}{M-2}\left(\frac{\frac{1}{M}\sum_{i=1}^{M}{(sgnl(i)-fst(1))}^{3}}{\frac{1}{M}\sum_{i=1}^{M}{(sgnl\left(i\right)-fs(1))}^{2}}\right)$$

20 $$fst(7)=\frac{M-1}{(M-2)(M-3)}\left[\left(M+1\right)\left(\left(\frac{\frac{1}{M}\sum_{i=1}^{M}{\left(sgnl(i)-fst(1)\right)}^{4}}{\frac{1}{M}\sum_{i=1}^{M}{\left(sgnl(i)-fst(1)\right)}^{2}}\right)-3\right)+6\right]$$

21 $$fst\left(8\right)=S(\lceil\frac{M}{2}\rceil)$$

22 $$fst\left(9\right)=\mathrm{min}(sgnl)$$

23 $$fst\left(10\right)=\mathrm{max}(sgnl)$$

24 $$fst\left(11\right)=\sum_{i=1}^{N}sgnl{\left(i\right)}^{2}$$

25 $$fs\left(12\right)=\sqrt{\frac{\sum_{i=1}^{N}sgnl{\left(i\right)}^{2}}{M}}$$

26 $$fst\left(13\right)=fst\left(10\right)-fst(9)$$

27 $$fst\left(14\right)=fst\left(10\right)-fst(1)$$

where $fst$ is a statistical feature vector with a length of 14, $sgnl$ denotes signal, and $M$ defines the length of the signal.

Steps of the presented feature generation model are given below.

- Step 1: Apply TQWT to the framed signal. To express TQWT, a function ($TQWT(.,.,.,.)$) is defined.
  28 $$S{B}^{k}=TQWT\left(sgnl,\mathrm{2,3},17\right), k=\{\mathrm{1,2},\dots ,18\}$$
  The parameters (Q = 2, r = 3, and J = 17) were chosen by using the trial and error method. These parameters are the best-resulted parameters according to experiments.
- Step 2: Generate features by using Led-Pattern $\left(led-pat\left(.\right)\right)$ and $ist(.)$ functions. Therefore, the feature generation is called as fused feature generation. This step defines the fused feature generation from the raw EEG signal frame.
  29 $$f{t}^{1}\left(j\right)=led-pat\left(sgnl\right), j=\{\mathrm{1,2},\dots ,512\}$$
  30 $$f{t}^{1}\left(512+k\right)=ist\left(sgnl\right), k=\{\mathrm{1,2},\dots ,14\}$$
  31 $$f{t}^{1}\left(526+k\right)=ist\left(led-pat\left(sgnl\right)\right), k=\{\mathrm{1,2},\dots ,14\}$$
  where $f{t}^{1}$ defines features extracted from the raw signal. As seen from Eqs. 28, 29, 30, three feature generation methods are used together. These are Led-Pattern, statistical feature generation, and statistical feature generation of the textural features (Led-Pattern features). At the same time, Eqs. 28, 29, 30 defines feature concatenation.
- Step 3: Apply fused feature generation to decomposed sub-bands (SB).
  32 $$f{t}^{h+1}\left(j\right)=led-pat\left(S{B}^{h}\right), j=\left\{\mathrm{1,2},\dots ,512\right\}, h=\{\mathrm{1,2},\dots ,18\}$$
  33 $$f{t}^{h}\left(512+k\right)=ist\left(S{B}^{h}\right), k=\{\mathrm{1,2},\dots ,14\}$$
  34 $$f{t}^{h}\left(526+k\right)=ist\left(led-pat\left(S{B}^{h}\right)\right), k=\{\mathrm{1,2},\dots ,14\}$$
- Step 4: Concatenate the feature extracted.
  35 $$X\left(\left(t-1\right)\times 540+z\right)=f{t}^{t}\left(z\right), z=\left\{\mathrm{1,2},\dots ,540\right\}, t=\{\mathrm{1,2},\dots ,9\}$$
  where $X$ is the final feature vector.

These four steps given above are defined as the proposed fused feature generator. By using these four steps, 10,260 features are extracted.

### RFIChi2 feature selector

The second phase of the proposed Led-Pattern and RFIChi2 (Tuncer et al. 2020) based model is feature selection with RFIChi2. RFIChi2 is a hybrid and iterative selector (Tuncer et al. 2021). RFIChi2 has two primary objectives. These are to use the effectiveness of both and ReliefF and Chi2 (Raghu and Sriraam 2018) and to select the optimal number of features automatically. ReliefF generates both positive and negative feature weights. However, Chi2 (Liu and Setiono 1995) cannot generate these type weights. Therefore, the threshold point should be detected to eliminate redundant features by using Chi2. Moreover, redundant features can be determined by using Chi2 easily. Negative weighted features are determined as redundant features by using ReliefF. Therefore, there is no threshold point detection problem in the ReliefF. Iterative Chi2 (IChi2) is used to select the optimal number of features automatically. Steps of the RFIChi2 selector are given below.

1. Apply ReliefF to generated features.
2. Remove/eliminate the negative weighted features.
3. Use Chi2 to obtain qualified indexes.
4. Select feature vectors using the generated qualified indexes. Herein, an iteration range is defined. For this work, this range is defined as [100,1000].
5. Calculate misclassification rates of the chosen 901 feature vectors using SVM classifier with tenfold cross-validation. In this step, the used loss function is parametric. In this work, we have utilized SVM classifier as both loss value generator/calculator and classifier.
6. Choose the optimal feature vector using the calculated misclassification rates.

### Classification

The last phase of the presented LEDPatNet19 is the classification. SVM (Vapnik 1998, 2013) classifier is considered as a classifier to calculate results. The used classifier is named Cubic SVM. Cubic SVM is a polynomial SVM. The used parameters of this SVM classifier are given as follows. Third degree polynomial kernel has been used and coding method is one-vs-one. We have selected automatic kernel scale for the used SVM. In Tables (in Tables 2–4), the best results are denoted/highlighted using bold font type.

## Results and discussion

### Experimental setup

We downloaded publicly available GAMEEMO and DREAMER datasets from the web^,^^1^. These databases have EEG signals of 23–28 subjects with 14 channels and includes two/four emotion classes. The used classes of the GAMEEMO dataset are funny, boring, horror and calm. The DREAMER dataset has three cases and these cases are arousal, dominance and valance. These cases have two classes and these classes are named low and high. The proposed LEDPatNet19 has been developed on these datasets. Moreover, MATLAB (2020) programming environment has been used with a computer to simulate our proposal.

### Experimental Results

The results of this model were given in this section. Two datasets (GAMEEMO and DREAMER) have been used to obtain results and to calculate measurements, accuracy, average recall ($AR$), average precision ($AP$), F1-score, and geometric mean were used. Mathematical expressions of these measurements were listed in Eqs. 36, 37, 38, 39

36 $$accuracy=\frac{t{p}_{c}+{tn}_{c}}{t{p}_{c}+{tn}_{c}+f{p}_{c}+f{n}_{c}}, c=\{\mathrm{1,2},\dots ,C\}$$

37 $$AR=\frac{1}{C}\sum_{c=1}^{C}\frac{{tp}_{c}}{{tp}_{c}+{fn}_{c}}$$

38 $$AP=\frac{1}{C}\sum_{c=1}^{C}\frac{{tp}_{c}}{{tp}_{c}+{fp}_{c}}$$

39 $$F1-score=\frac{2 \times AP \times AR}{AP+AR}$$

where ${tp}_{c},{fn}_{c},{tn}_{c}$ and ${fp}_{c}$ are true positives, false negatives, true negatives and false positives of the c^th^ class. In the classification problem, four classes were used. The proposed LEDPatNet19 uses RFIChi2 feature selector. This selector chooses variable number of features per the used problem. The selected number of features for each channel according to the dataset/case are tabulated in Table 1.

**Table 1:** The number of selected features for each channel using RFIChi2

Channel	DREAMER			GAMEEMO
	Arousal	Dominance	Valance	
AF3	879	731	806	974
AF4	451	602	745	890
F3	988	422	890	780
F4	566	351	631	969
F7	580	448	842	937
F8	707	805	848	898
FC5	402	546	547	856
FC6	536	616	702	862
O1	548	463	834	932
O2	710	313	960	835
P7	684	373	648	931
P8	717	407	991	824
T7	748	242	976	677
T8	710	882	625	989

The selected feature vectors (length of the selected feature vectors are listed in Table 1). These feature vectors are fed to Cubic SVM classifier. tenfold cross-validation have also been utilized as validation technique. The calculated results of the proposed LEDPatNet19 per the dataset/case are denoted in Tables 2 and 3.

**Table 2:** The obtained performance rates for GAMEEMO and DREAMER arousal case

Channel	GAMEEMO	DREAMER/arousal						
	Acc	Rec	Pre	F1	Acc	Rec	Pre	F1
AF3	98.75	98.75	98.75	98.75	91.19	89.57	91.87	90.71
AF4	98.57	98.57	98.58	98.58	**94.58**	**93.47**	**95.15**	**94.30**
F3	99.11	99.11	99.11	99.11	90.51	88.21	92.21	90.16
F4	98.39	98.39	98.41	98.40	91.86	89.80	93.46	91.59
F7	98.21	98.21	98.24	98.23	89.83	87.65	91.12	89.35
F8	98.75	98.75	98.76	98.75	91.86	89.96	93.16	91.53
FC5	98.57	98.57	98.59	98.58	88.14	86.27	88.57	87.41
FC6	**99.29**	**99.29**	**99.30**	**99.29**	88.47	85.74	90.48	88.05
O1	99.11	99.11	99.11	99.11	88.14	85.30	90.25	87.70
O2	98.39	98.39	98.41	98.40	89.15	87.10	90.07	88.56
P7	98.57	98.57	98.58	98.57	89.49	87.22	90.88	89.01
P8	98.57	98.57	98.59	98.58	89.83	87.49	91.42	89.41
T7	98.04	98.04	98.05	98.04	89.49	86.73	91.88	89.23
T8	98.57	98.57	98.58	98.57	90.17	87.61	92.32	89.90

*Acc* Accuracy, *Rec*: Recall, *Pre* Precision, *F1* F1-score

**Table 3:** The obtained performance rates for DREAMER dominance and DREAMER valance cases

Channel	DREAMER/dominance	DREAMER/valance						
	Acc	Rec	Pre	F1	Acc	Rec	Pre	F1
AF3	89.46	84.78	91.14	87.85	91.98	91.97	91.98	91.98
AF4	**92.86**	**89.50**	**94.32**	**91.85**	92.90	92.90	92.90	92.90
F3	89.80	85.31	91.38	88.24	91.67	91.66	91.68	91.67
F4	89.12	84.53	90.47	87.40	90.12	90.12	90.12	90.12
F7	87.76	81.88	90.49	85.97	**94.44**	**94.43**	**94.63**	**94.53**
F8	92.52	88.97	94.09	91.46	93.83	93.83	93.83	93.83
FC5	87.41	81.90	89.24	85.42	92.28	92.27	92.33	92.30
FC6	87.07	81.93	88.12	84.91	91.98	91.97	92.01	91.99
O1	85.71	80.65	85.91	83.19	87.35	87.35	87.35	87.35
O2	89.46	85.06	90.71	87.80	92.59	92.60	92.61	92.61
P7	89.80	85.86	90.57	88.15	91.05	91.05	91.05	91.05
P8	90.82	86.89	92.09	89.42	90.74	90.73	90.77	90.75
T7	88.78	84.56	89.43	86.93	92.90	92.89	92.95	92.92
T8	90.82	86.34	92.98	89.54	91.98	91.96	92.15	92.05

As can be seen from Table 2, the best accuracy rates of the GAMEEMO and DREAMER/arousal datasets have been achieved 99.29% and 94.58% respectively. The best resulted channel for GAMEEMO dataset is FC6 and AF4 is the best channel for DREAMER/arousal. Furthermore, the best results are denoted using bold font type and accuracy and overall recall value are the same for GAMEEMO dataset since this dataset is a homogenous dataset. Results of the DREAMER/dominance and DREAMER/valance problems are tabulated in Table 3.

By using our proposed LEDPatNet19, 92.86% and 94.44% classification accuracies have been calculated on the DREAMER/dominance and DREAMER/valance cases respectively. The best results have been calculated using AF4 and F7 channels consecutively.

### Discussions

**Fig. 4:**
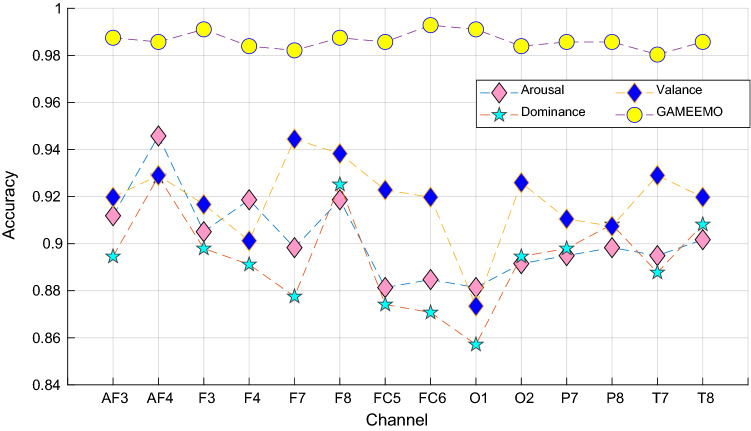
Channel-wise classification accuracies of the proposed LEDPatNet19 per the used datasets

This research presents a new emotion classification model by using EEG signals. The presented model uses a nonlinear textural feature generator, which is called Led-Pattern. By using TQWT, Led-Pattern, and statistical features (14 statistical moments), a fused multilevel feature generation network is presented. RFIChi2 selects the most discriminative features, which are utilized as an input of SVM classifier. This model is tested on publicly available GAMEEMO and DREAMER EEG datasets. These datasets have 14 channeled EEG signals. Mainly, RFIChi2 selected a variable number of features for each channel. The reached classification accuracies have been tabulated in Tables 2and 3. The proposed LEDPatNEt19 attained 99.29% on the GAMEEMO dataset and 94.58% accuracy rate on the arousal case of the DREAMER database. Channel-wise results of the proposed LEDPatNEt19 according to the used dataset are also denoted in Fig. 4.

To obviously illustrate the success of the proposed LEDPatNet19 emotion recognition model, this model was compared to other emotion recognition method using the GAMEEMO dataset. These results were listed in Table 4.

**Table 4:** Accuracy rates (%) for the multiclass classification of the Alakus et al.’s method and our presented Led-Pattern and RFIChi2method

Method	AF3	AF4	F3	F4	F7	F8	FC5	FC6	O1	O2	P7	P8	T7	T8
Alakus et al.’s method + kNN (lakus et al. (2020)	42	55	35	43	43	54	47	36	43	38	41	40	38	45
Alakus et al.’s method + SVM Alakus et al. (2020)	54	50	40	54	70	69	34	34	55	54	66	70	47	79
Alakus et al.’s method + MLPNN Alakus et al. (2020)	80	75	75	82	71	71	75	74	71	65	70	72	65	79
LEDPatNet19	98.75	98.57	99.11	98.39	98.21	98.75	98.57	**99.29**	99.11	98.39	98.57	98.57	98.04	98.57

As it can be seen from Table 4, the best results of the Alakus et al.’s method were achieved on MLPNN classifier. Our best result is greater 19.29% greater than the best result of the Alakus et al.’s method.

In order to better imply the success of the suggested LEDPatNet19 for DREAMER dataset, comparative results are tabulated in Table 5.

**Table 5:** Comparative results for DREAMER dataset

Study	Method	Accuracy (%)		
		Arousal	Dominance	Valance
Cheng et al. (2020)	Deep neural networks	90.41	89.89	89.03
Bhattacharyya et al. (2020)	Fourier–Bessel series expansion based empirical wavelet transform	85.40	84.50	86.20
Li et al. (2021)	3-D feature representation and dilated fully convolutional networks	79.91	80.23	81.30
Liu et al. (2021)	Deep canonical correlation analysis	89.00	90.70	90.60
Wang et al. (2021b)	Frame-level distilling neural network	87.67	90.28	89.91
Wang et al. (2021a)	Domain adaptation symmetric and positive definite matrix network	76.57	81.77	67.99
Zhang et al. (2021)	Generative adversarial networks	94.21	–	93.52
Galvão et al. (2021)	Wavelet energy and entropy	93.79	–	93.65
Our method	LEDPatNet19	94.58	92.86	94.44

According to Table 5, the results obtained show the performance of the proposed method for the DREAMER dataset. LEDPatNet19 attained the best classification rates among the developed state of art methods. Moreover, Cheng et al. (Cheng et al. 2020) proposed a deep learning based EEG emotion classification model and our proposed LEDPatNet19 also attained better results than deep model. Advantages of the proposed LEDPatNet19 are:

- Effectiveness of the Led-Pattern, which is a nonlinear pattern, for EEG-based emotion recognition was demonstrated. This research demonstrated a new generation hand-crafted feature generation study area, which is named as S-Box based nonlinear textural feature generation.
- This research aimed to eliminate two fundamental problems, which are feature extraction and feature selection problems. By using a multilevel fused feature generation network (TQWT, statistical features, and the presented Led-Pattern), feature extraction problem is solved. Moreover, RFIChi2 solved the feature selection problem. Since it uses ReliefF and iterative Chi2 together, it has automated the optimum number of features selection.
- By using SVM classifiers (it is a shallow classifier), high classification accuracies were obtained for all channels (see Tables 2 and 3).
- The proposed LEDPatNet19 emotion classification model has achieved better performance than the previous studies that uses the same dataset (see Tables 4 and 5).
- General classification accuracy of the proposed LEDPatNet19 is able to achieve high classification accuracy on the two EEG emotion datasets.

## Conclusions and future directions

This work presents a new generation emotion recognition model. This model has four fundamental phases, which are preprocessing, fused feature generation using Led-Pattern and statistical feature generator, discriminative features selection by RFIChi2, and classification using SVM. Our presented LEDPatNet19 model was able to over 92% classification accuracies for the used GAMEEMO and DREAMER datasets. These results clearly demonstrate the success of the presented emotion recognition model. Also, the presented LEDPatNet19 is compared to other emotion classification models and achieved better performance. The proposed model can be used for developing an automated emotion recognition method during game playing, driving fatigue detection, and seizure prediction and detection in future works. We are planning to develop a new generation nonlinear S-box pattern-based deep network. The new nonlinear patterns can be presented by using other S-boxes.

## Footnotes

1 https://data.mendeley.com/datasets/b3pn4kwpmn/1. https://zenodo.org/record/546113#.YUioBLgzaUk.
